# Current Knowledge of Enterococcal Endocarditis: A Disease Lurking in Plain Sight of Health Providers

**DOI:** 10.3390/pathogens13030235

**Published:** 2024-03-07

**Authors:** Francesco Nappi

**Affiliations:** Department of Cardiac Surgery, Centre Cardiologique du Nord, 93200 Saint-Denis, France; francesconappi2@gmail.com or f.nappi@ccn.fr; Tel.: +33-149-334-104; Fax: +33-149-334-119

**Keywords:** *Enterococcus faecalis*, vegetation, infective endocarditis, biofilm, cardiac endovascular infection, gastrointestinal

## Abstract

*Enterococcus faecalis* is a bacterial pathogen that can cause opportunistic infections. Studies indicate that initial biofilm formation plays a crucial regulatory role in these infections, as well as in colonising and maintaining the gastrointestinal tract as a commensal member of the microbiome of most land animals. It has long been thought that vegetation of endocarditis resulting from bacterial attachment to the endocardial endothelium requires some pre-existing tissue damage, and in animal models of experimental endocarditis, mechanical valve damage is typically induced by cardiac catheterisation preceding infection. This section reviews historical and contemporary animal model studies that demonstrate the ability of *E. faecalis* to colonise the undamaged endovascular endothelial surface directly and produce robust microcolony biofilms encapsulated within a bacterially derived extracellular matrix. This report reviews both previous and current animal model studies demonstrating the resilient capacity of *E. faecalis* to colonise the undamaged endovascular endothelial surface directly and produce robust microcolony biofilms encapsulated in a bacterially derived extracellular matrix. The article also considers the morphological similarities when these biofilms develop on different host sites, such as when *E. faecalis* colonises the gastrointestinal epithelium as a commensal member of the common vertebrate microbiome, lurking in plain sight and transmitting systemic infection. These phenotypes may enable the organism to survive as an unrecognised infection in asymptomatic subjects, providing an infectious resource for subsequent clinical process of endocarditis.

## 1. Introduction

**Figure 1:**
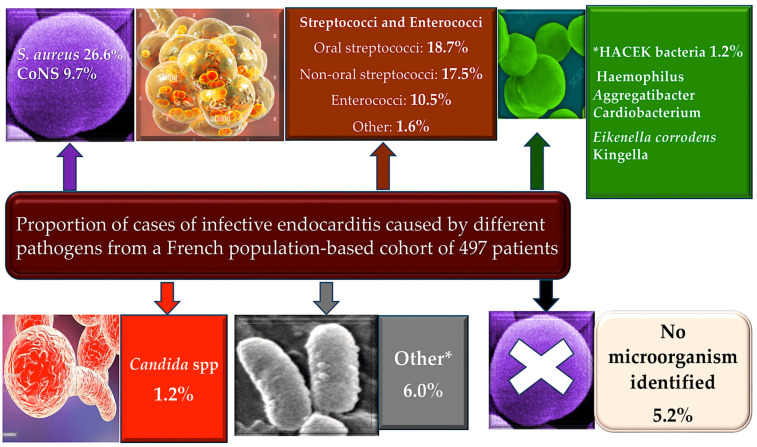
Elderly patients with a history of CIED and younger patients with a history of PWID have a higher incidence of IE. Low incidence of IE in patients with central venous catheters, HIV, CHD, and immunosuppression. In total, 26.6% of IE cases occur due to *Staphilococcus aureus* and 9.7% of these occur due to CoNS. Enterococci are involved in more than 10% of cases. Zoonotic endocarditis is determined by *Coxiella burnetii* and *Brucella* (from livestock), *Bartonella henselae* (from cats), and *Chlamydia psittaci* (from parrots, pigeons). Other rare causes include Gram-negative bacteria (e.g., *Acinetobacter* spp., *Pseudomonas aeruginosa*), *Legionella* spp., *Mycoplasma* spp., and *Tropheryma whippelii*. Fungal endocarditis, usually *Candida* or *Aspergillus*, is rare but often fatal, arising in patients who are immunosuppressed or after cardiac surgery, mostly on prosthetic valves. From Nappi et al. [12,13]. * Classification from Lancet.

Enterococci are a unique type of bacteria due to their ability to withstand a broad range of different environmental parameters such as pH, temperature, salinity, bile acids, and so on. They are resistant to many antibiotic compounds and have the flexibility to flourish as both common commensal and opportunistic pathogens in a broad range of clinical settings [1,2,3,4,5,6]. Enterococci commonly live in the body and can cause chronic endocarditis, especially *Enterococcus faecalis* [5,7,8,9,10]. They account for approximately 10% of valvular endocarditis cases, with *E. faecalis* being the main causative agent [6,8,9,10,11,12,13] (Figure 1).

To improve patient outcomes, it is important to accurately diagnose and treat enterococcal infections. During colonisation of the murine gastrointestinal (GI) tract, *E. faecalis* has been shown to form and develop bacterial biofilms. These biofilms consist of bacteria attached to a host surface and surrounded by a bacterially derived extracellular matrix (ECM) [13,14]. In animal models of enterococcal catheter-associated urinary tract infections and endocarditis, *E. faecalis* has been identified as a significant pathogenic factor [15,16,17,18,19,20,21,22]. This finding was first reported in 2007 [22]. Colonisation results in the formation of a defensive bacterial biofilm on the native or engineered tissue: biofilm formation often results in markedly enhanced levels of resilience to antimicrobial agents [23,24,25].

Bacteria colonise a prestaged, abacterial collection of host factors according to the classical or canonical model of bacterial endocarditis. The model proposes a two-step process: First, platelets, components of the coagulation chain including fibrinogen, thrombin, etc., and other host factors are deposited in reaction to an initial injury, thereby creating a “sterile vegetation”. Bacteria already circulating in the bloodstream then populate this aberrant site, establishing a largely quiescent infection nidus [11]. Likewise, enterococcal infection is a significant cause of dysfunction in allogeneic tissue used as a biological valve replacement for patients who have received an allograft, whether for endocarditis or non-endocarditis of the aortic valve [12,26]. 

Barnes et al. [14] have previously reported that *E. faecalis* directly engages and colonises the surface of the intestinal epithelium, producing distinct biofilm microcolonies across the gastrointestinal tract in a germ-free mouse model of infection. In a rabbit model of cardiac endovascular infection, a comparable pattern of colonisation of the native host surface is also observed [13]. These observations suggest that the adhesion of enterococci to the cardiac endothelium has a similar role in the development of pathogenic endocarditis as it does in non-pathogenic intestinal epithelial colonisation. This is supported by the absence of significant systemic host responses to this colonisation over several weeks and the ability of *E. faecalis* to adhere to intact endothelia.

This section reviews both the current and past findings for this kind of infection, shows how the conventional model fits, and fails to fit, with recent findings in the area, and considers possible future directions for better understanding the pathophysiology of this increasingly important clinical infection (Table 1). The infection rate was assessed in patients who received an allograft, both those who underwent surgery for aortic valve endocarditis and those who underwent surgery for reasons unrelated to infection. The causative pathogen type was investigated in previous cardiac surgery and reoperation. 

## 2. History

Originally described in the early 20th century and named *Streptococcus faecalis* before being placed in the genus *Enterococcus* in 1984, *Enterococcus faecalis* has been known to cause endocarditis since the seminal paper published by Andrewes and Horder in 1906 [27].

As previously mentioned, the conventional paradigm for bacterial colonisation of the heart involves an abiotic accumulation of host factors. This is usually accompanied by an endovascular injury. Nevertheless, it is noteworthy that numerous papers in the earlier literature (prior to 1975) reported that enterococcal endocarditis appeared to arise in a substantial proportion of individuals without obvious prior gross endothelial damage or structural cardiac defects [28,29]. As is frequently the case in earlier literature, the exact determination of the particular bacterial strain can be challenging. Several animal model models, notably pigs [30] and rabbits [31], have also described these clinical findings. 

During the 1970s and 1980s, the medical community focused on enterococci because of their high level of intrinsic and transmissible antibiotic resistance in comparison to pathogenic streptococci. It is worth noting that until the 1980s, enterococci were phylogenetically classified as members of the genus *Streptococcus* [32]. 

The interest of the medical and health care community in enterococci during the 1970s and 1980s was largely driven by the relatively high level of inherent and transmissible antibiotic resistance of these bacteria compared to the pathogenic streptococci routinely found in the population. It is noteworthy to mention that enterococci were classified phylogenetically as belonging to the species *Streptococcus* right up to the 1980s [32]. During this time, genetic and molecular studies of both plasmids and trans-spliced genetic material provided an important experimental basis for future genomic approaches to enterococcal virulence [33,34]. Yet, the global clinical frequency of clinically ascertained enterococcal infections continued to be low throughout much of this time frame, although it is unclear whether this represents a real incidence rate or merely a reflection of a more restricted diagnostic landscape.

In the 1980s, the widespread use of oral prophylaxis with cephalosporins led to the emergence of enterococci (mainly *E. faecalis*) as the most important hospital pathogens. Certain genotypes were able to achieve epidemic dissemination, both nationally and internationally [35,36,37,38,39,40,41]. Starting in the 1990s, systematic attempts to determine crucial genetic factors of virulence in nosocomial and other opportunistic enterococcal infections were intensified as a result of these clinical developments. Pioneering studies in this field aimed to identify enterococcal antigens that triggered an antibody response in patients with infections [42,43,44,45,46,47,48,49,50]. In early studies, most of the prominent antigens discovered were surface-exposed antigens of the enterococcal cell coat (Ebp, Ace, Epa). Subsequent studies using in vitro assays and animal models, including experimental endocarditis, have identified critical roles for these constituents in host adherence and virulence [42,43,44,45,46,47,48,49,50]. In addition to the factors mentioned above, which are genetically determined, there is also evidence that plasmid-encoded surface adhesins, such as Aggregation Substance, play a role [51,52,53,54,55,56,57]. 

Enterococci have become increasingly significant in healthcare-associated infections over the last two decades. This trend is likely to be driven by a number of factors. Among them are increased access to diagnostics, an increasingly elderly population, greater invasiveness of medical interventions, and the continued emergence of antimicrobial resistance [58,59,60,61,62,63]. During this time period, the number of studies in the general area of bacterial biofilms increased markedly [64,65,66,67,68,69,70,71,72]. Additionally, the full genome sequence for *E. faecalis* V583 was published [73,74,75]. In 2003, Bourgogne et al. [76] identified OG1RF, and since then, several other strains have been extensively studied [77] using enhanced genetic research tools to investigate *E. faecalis* infection [78,79,80,81,82]. Our knowledge of the genetic basis of biofilm development in *E. faecalis*, both during in vitro propagation and infection, has been greatly enhanced as a result [9,83,84,85,86]. Barnes et al. [13,14] conducted a thorough study on transposon mutagenesis and recombinase-based in vivo expression technology (RIVET) genetic screens. The results were non-overlapping but mutually supportive, identifying several factors involved in multiple in vitro biofilm production in the chromosome of strain OG1RF. These findings were previously reported by Kristich et al. [80] and Ballering et al. [81]. When the same RIVET library was tested in a rabbit model of subcutaneously implanted foreign body infection, 28 genes identified in these in vitro tests (two from the transposon screen, 26 from the RIVET screen) were also found to have promoters [19]. However, only two genes (ahrC and eep) were considered to play a significant role in endocarditis pathogenesis when ten strains with mutations in biofilm-associated genes from these candidate genes were tested for in vivo virulence impairment in a rabbit model of infective endocarditis [19,87]. Leuck et al. [88] found that *E. faecalis* clinical strains that were classed as poor biofilm producers in a standard in vitro microtiter dish assay colonised porcine heart valves in an ex vivo assay just as well as strong biofilm-forming clinical strains, supporting the conclusion that in vitro biofilm phenotypes do not closely predict infective endocarditis.

**Figure 2:**
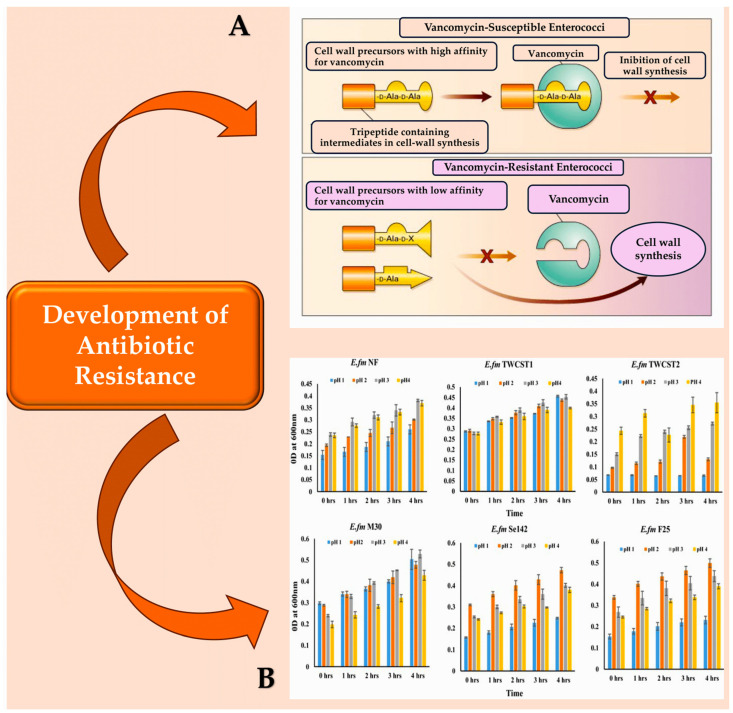
(**A**): Vancomycin-susceptible enterococci synthesise cell wall precursors that bind vancomycin with high affinity. These precursors end in D-Ala-D-Ala and are translocated from the cytoplasm to the cell surface where, once bound, they cannot participate in cell wall synthesis. In the presence of an inducer like vancomycin, vancomycin-resistant enterococci produce intermediates with different end groups (D-Ala-D-Lac, D-Ala, or D-Ala-D-Ser), which have a low affinity for vancomycin and can therefore be used for cell wall synthesis. (**B**): The resistance of the chosen enterococcal strains to artificial gastric juice, containing pepsin and acidified to pH 1.0 to pH 4.0, was tested by incubating them for 4 h at 37 °C. This mimics the transient time food spends in the stomach. Abbreviations: ‘LA’ denotes either alanyl or alanine, while ‘X lactate’ is used for VanA, VanB, and VanD types of resistance, and ‘serine’ is used for VanC and VanE types [91,92,93,94].

Madsen et al. conducted a systematic literature review that summarised nine virulence factors of *E. faecalis* infective endocarditis [17]. This information is highly useful for readers. The virulence factors listed therein comprise the aggregation substance, cell wall glycolipids, the Ebp pili proteins, haemolysin, the stress protein gls24, the secreted protease GelE, the membrane metalloprotease Eep, and the adhesins Ace and EfbA [17,89]. The transcriptional regulator AhrC is the tenth virulence factor of *E. faecalis* endocarditis. It affects the expression of the ace and ebp genes, as reported by Frank et al. [19] and Manias and Dunny [54,85,90] (Figure 2 [91,92,93,94]).

The genetic drivers involved in *E. faecalis* biofilm formation are shown in Table 2 [83]. Table 2 Shows the genetic determinants that are involved in the formation of *E. faecalis* biofilm [47,83].

## 3. Causes of *E. faecalis* Bacteraemia

**Figure 3:**
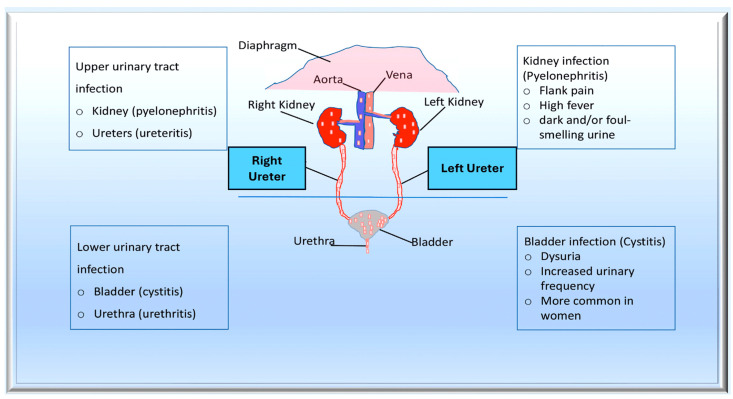
The pathogenesis of urinary tract infections (UTIs) begins with the colonisation of uropathogens in the urethra, followed by the bladder, facilitated by specific adhesins. Causative bacteria proliferate and form biofilms if they are not eliminated by the immune system. Pathogens can ascend from the lower urinary tract to the kidney, leading to bacteraemia. Uropathogens can bind to the catheter and multiply within the biofilm in the case of complicated UTIs. The infection may progress to pyelonephritis and bacteraemia if left untreated. From Mancuso et al. [99].

Bacteraemia is evidently required for endothelial bacterial colonisation of the endothelium and the development of IE. In cases of acute bacteriaemia, the initial source of infection is often identifiable. This is due to the short period of time between the spread of bacteraemia and the onset of IE. Chronic endocarditis, which is similar to the classic enterococcal endocarditis, is often much more ambiguous [96,101]. A variety of causes have been proposed, varying from colonisation of the oral cavity in endodontic disease to translocation of commensal enterococci in the gastrointestinal tract [97,98]. *Enterococcus* is the second leading cause of hospital-acquired bacteraemia, due in part to its ability to thrive in challenging environments. Contamination of environmental surfaces in healthcare settings can cause exogenous infection, leading to direct seeding of the vasculature through catheterisation or contamination of implantable medical devices. Indirect infection can also occur through colonisation of the urinary or gastrointestinal tracts. Endogenous infections can also result from translocation through the epithelium of the GI tract [97,98,99,100,102,103] (Figure 3).

This process is facilitated by conventional antibiotic regimens, which can drastically increase the number of enterococci in the intestinal flora [104,105]. More than three decades ago, Wells et al. [106] experimentally demonstrated translocation of *E. faecalis* across the epithelial barrier of the GI system and subsequent penetration into the circulation in a mouse model. More advanced work has followed, including detection of invasion-defective *E. faecalis* mutant strains in a T84 cell culture model [107,108,109] and high-resolution imaging of the process with complementary findings on intracellular migration [110]. Despite the long-standing belief that oral enterococci are a likely source for endocarditis, cohort evidence has shown that oral infections are not a common factor in IE, despite the fact that enterococci are also commonly found in the oral cavity and are a leading etiology of endodontic disease [110]. For instance, only 1.6% of enterococcal cases could be attributed to oral routes of transmission versus 6.7% of non-enterococcal cases in a recent large Spanish cohort study comparing enterococcal IE (516 patients) and non-enterococcal IE cases (3308 patients) [59].

Severe physiological challenge, in combination with the possibility of organism-specific translocation, may result in enough GI barrier breakdown to permit bacterial penetration via systemic host immunosuppression [111,112,113,114]. It is unclear whether enterococcal translocation is a result of host immunosuppression or if enterococci themselves are immunomodulatory and can initiate the suppressive response [112]. In a mouse model, common antibiotics at clinically relevant doses can cause GI barrier dysfunction and bacterial translocation, in some cases after a single dose. Again, *E. faecalis* is a key player [115,116,117]. 

Brown et al. [118] have recently reported the discovery of cardiac microlesions during severe bacteraemia caused by *E. faecalis* infection in mice. These microinjuries are similar to those caused by *Streptococcus pneumoniae* during invasive pneumococcal disease. However, *E. faecalis* does not encode the virulence determinants involved in pneumococcal microinjury formation. The study discovered that the protein DsbA, which forms disulphide bonds, is essential for *E. faecalis* virulence in a *C. elegans* model and for the efficient formation of cardiac microlesions. Additionally, *E. faecalis* facilitated necroptotic cell death of cardiomyocytes at sites of microlesion formation. Unlike the wild-type strain, which suppressed the immune response, loss of DsbA resulted in an increase in pro-inflammatory cytokines. Furthermore, *E. faecalis* was able to induce microlesions in the heart. This study has identified the features of both the bacterium and the host response that are involved in this process. 

Although there is only a paucity of clinical evidence to date, there is also some emerging data on an association between enterococcal endocarditis events and cryptic colorectal cancers [119,120,121]. It is uncertain whether there is a significant association between these clinical conditions, as seen in most cases of *Streptococcus gallolyticus* subsp. gallolyticus endocarditis, previously associated with *Streptococcus bovis* biotype I [122,123,124,125,126]. Stanley et al. [127] found that a murine model of ischemic-reperfusion stroke showed bacteraemia caused by a specific group of commensal bacterial strains, with enterococci being the most prevalent.

### 3.1. Induced Enterococcal Colonisation Involves Cell Surface Mechanisms—Ultra-Large von Willebrand Factor and Sortase Are Key Players in This Process

The accepted developmental pathway for bacterial endocarditis includes the primary production of a host-derived thrombus, with subsequent processes promoting colonisation of the thrombus by bloodstream bacteria. However, there are multiple instances where direct colonisation of host epithelial surfaces has been reported and in practice, this mode of adhesion may be more prevalent than is currently recognised. *S. aureus* is one of the most studied of those bacterial pathogens that have been demonstrated to directly adhere to the endothelium, at least under some circumstances. *S. aureus* expresses three fundamental molecules on its surface: fibronectin-binding protein A (FnBPA) and B (FnBPB), as well as clumping factor A (ClfA). These molecules promote bacterial adherence and identify the cultured human endothelial cells (ECs) that interact with Gram-positive cocci. Three recent reports have investigated the adherence of Gram-positive cocci to endothelial cells (ECs) and have highlighted the fundamental importance of these molecules in IE [128,129,130]. 

Pappelbaum et al. [131] showed that *Staphylococcus aureus* adhesion to healthy endothelial cells is associated with elevated levels of ultra-large von Willebrand factor, a host cofactor that deserves in-depth analysis due to its peculiarities of action. Bacterial proteins, such as ClfA and FnBPA, help *S. aureus* stick to EC surface molecules. This is also done by subendothelial matrix proteins, like fibrinogen, fibrin, fibronectin, and von Willebrand factor (vWF). In the setting of the undamaged endothelium, evidence suggests that ultra-large von Willebrand factors (ULVWFs) significantly facilitate the initial pathogenic phase of *S. aureus*-induced endocarditis. When activated human endothelial cells were perfused with fluorescent bacteria under high-shear-rate conditions, 95% of the *S. aureus* attached to ultra-large von Willebrand factor (ULVWF) [131]. Flow experiments using VWF deletion mutants and heparin indicated that the A-type domains of VWF contribute to bacterial binding. The role of wall teichoic acid, but not staphylococcal protein A, was suggested by analysis of several bacterial deletion mutants. ULVWF-mediated bacterial adherence significantly increased with the presence of inactivated platelets and serum. ADAMTS13, a thrombospondin 13 disintegrin and metalloproteinase, reduced bacterial binding and shortened the length of ULVWF in a dose-dependent manner, but even at physiological levels of ADAMTS13, individual cocci remained bound by ULVWF. To further demonstrate the role of VWF in vivo, wild-type mice were compared with VWF knockout mice. Using the dorsal skinfold chamber model and intravital microscopy, fluorescent bacteria binding was observed in tumour necrosis factor-α-stimulated tissue. VWF knockout mice had fewer bacteria in their postcapillary and collecting venules compared to wild-type mice. Using heparin and ADAMTS13 can reduce ULVWF formation and may provide a novel therapeutic option to prevent IE [131].

**Figure 4:**
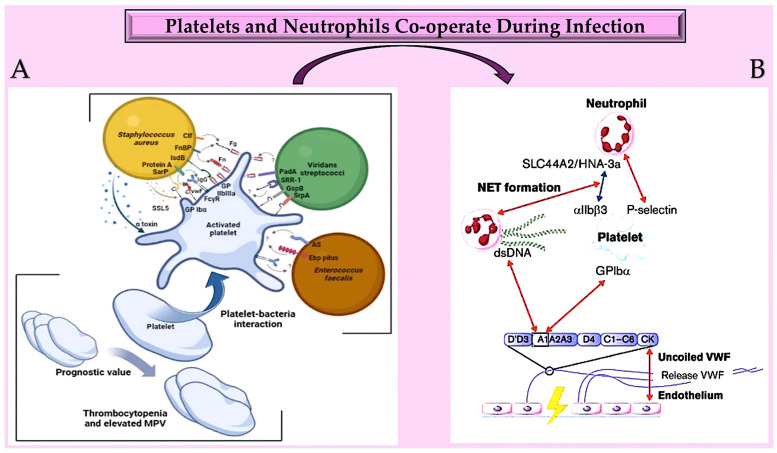
(**A**) Platelets play a crucial role in infective endocarditis. The interactions between platelets and the bacterial species involved in IE are complex and involve numerous ligand–receptor pairs. Changes in platelet parameters have predictive value. Additionally, von Willebrand factor is also involved. (**B**): The diagram illustrates the interactions between von Willebrand factor (VWF) and neutrophils at an infection site. It provides insights into the relationships between the A1 domain of VWF multimers, platelets, neutrophils, and NETs under conditions of high and low shear flow (indicated by red and blue arrows, respectively). Abbreviations: AS, aggregation substance; Clf, clumping factor; Ebp, endocarditis- and biofilm-associated pili; FcγR, crystallisable fragment gamma receptor; Fg, ds, double strand; fibrinogen; Fn, fibronectin; FnBP, fibronectin-binding protein; GP, glycoprotei; GspB, *Streptococcus gordonii* surface platelet B; IgG, immunoglobulin G; IsdB, iron-responsive surface determinant B; PadA, platelet adherence protein A; Sar P, staphylococcal accessory regulator protein; SrpA, serine-rich glycoprotein A; SRR-1, serine-rich repeat glycoprotein 1; SSL5, staphylococcal superantigen-like 5; VWF, von Willebrand factor [128,129,130,131,132,133,134,135,136,137,138,139,140,141,142,143,144].

Research has been conducted on the cell biology of NETosis in the context of infection [132]. The enzyme PAD4, which stands for protein arginine deiminase 4, plays a crucial role in this process. PAD4 is the only member of the PAD family that possesses a nuclear localisation signal [133,134,135,136]. Furthermore, it is believed that PAD4 has particular targets within the cytoplasm that affect the cell biology of NETosis and the composition of the neutrophil inflammasome. During an infection, functional cytoplasts (enucleated cells) capable of supporting phagocytosis can be identified. In blood vessels, NETs act as a platform for platelet adhesion and initiation of coagulation, similar to VWF [134,135,137,138]. Active PAD4, which is released in conjunction with NETs, also facilitates the citrullination of ADAMTS13. This impedes VWF scission and allows platelet aggregates to remain close to the vessel wall in the presence of PAD4 [139,140]. Recent studies have linked NETosis and the increase in NET-associated tissue factor (TF) to systemic inflammation and IL-1β levels, indicating a common regulatory pathway [141]. Additionally, TF secretion from activated macrophages and monocytes is stimulated by the activation of both canonical and non-canonical inflammasomes, as demonstrated by recent research [142,143] (Figure 4).

The role of vWbp and the sortase-assembled pilus family emerged during the analysis of adhesion mechanisms in Gram-positive cocci infections. Claes et al. [128] discovered that the interaction between vWbp and surface proteins of *S. aureus* reduces bacterial adhesion to VWF and vascular endothelium under shear stress. Mutants deficient in Sortase A (SrtA) and SrtA-surface proteins, as well as *Lactococcus lactis*-transmitting single staphylo-surface proteins, have been employed. S. aureus attaches to the endothelium via vWF. The VWF-binding protein (vWbp) facilitates adhesion under shear stress. The vWbp interacts with vWF to complete the adhesion process. It is suggested that the synergistic action of Sortase, a ClfA-dependent surface protein, plays a role in this process. 

Similarly, *Enterococcus faecalis* is an opportunistic bacterium that causes various hospital-acquired infections, including catheter-associated urinary tract infections. It may contribute to virulence and the development of infective endocarditis. In a mouse model of *E. faecalis*-ascending urinary tract infections, the role of the endocarditis- and biofilm-associated pilus (Ebp), a member of the sortase-assembled pilus family, was demonstrated. The Ebp pilus consists of the major EbpC shaft subunit and the minor subunits EbpA and EbpB. In experimental catheter-associated urinary tract infections, the EbpABC(-) strain, a non-piliated pilus knockout mutant, was significantly less virulent than its isogenic parent OG1RF. In contrast, the EbpC(-) strain, which is a mutant with a deleted nonpiliated ebpC gene, exhibited similar behaviour to OG1RF in vivo because it expressed EbpA and EbpB. Deletion of either the minor pilin gene ebpA or ebpB disrupted pilus biogenesis and resulted in defects in experimental catheter-associated urinary tract infections. The Ebp pilus has been identified as a virulence factor in *E. faecalis* catheter-associated urinary tract infections. Its in vivo function depends on a metal ion-dependent adhesion site motif that is predicted in EbpA’s von Willebrand factor A domain. Understanding the molecular basis of this common protein domain among the tip subunits of sortase-assembled pili is important in preventing and treating catheter-associated urinary tract infections caused by *Enterococcus faecalis*. The Ebp pilus of *E. faecalis* and its subunits are crucial to the virulence of enterococcal infections in a mouse model of catheter-associated urinary tract infections. The metal ion-dependent adhesion site motif in EbpA is crucial for Ebp function in vivo. This discovery has implications for the molecular basis of virulence in *E. faecalis* catheter-associated urinary tract infections, as well as other infections caused by enterococci and other Gram-positive pathogens. The metal ion-dependent adhesion sitemotif is also present in other sortase-assembled pili [128]. 

### 3.2. The Role of the Endocardium and Enterococcal Pathoadaptation

The endothelium is a specialised type of epithelium. This concept offers an intriguing explanation. Several studies have confirmed that the endocardium is indeed a modified endothelium [145,146,147,148,149], although there has been some uncertainty about the specifics of endocardial development. *E. faecalis* can directly colonise different host epithelial surfaces in a variety of animal experimental models. In a germ-free mouse model, Barnes et al. [14] demonstrated that *E. faecalis* can successfully colonise the surface of the intact, normal intestinal epithelium directly. Barnes et al. [13] have recently suggested that enterococcal coverage of endocardial and endovascular surfaces is possible without the need for host tissue destruction or even restricted surgical intervention, using a rabbit model of endocarditis. 

Endocarditis caused by *E. faecalis* is a serious clinical manifestation, commonly acquired in a community setting. Understanding the extrinsic pathogenesis at the valve level is a priority. Infective endocarditis is a complex disease with many host and microbial components contributing to the formation of bacterial biofilm-like vegetations on the aortic valve and adjacent areas of the heart. Thurlow et al. [21] reported further evidence supporting a non-valvular role in early endocardial colonisation. In their model, even after the inflamed valve was harvested, cardiac tissue homogenates still showed greatly elevated bacterial loads. 

**Figure 5:**
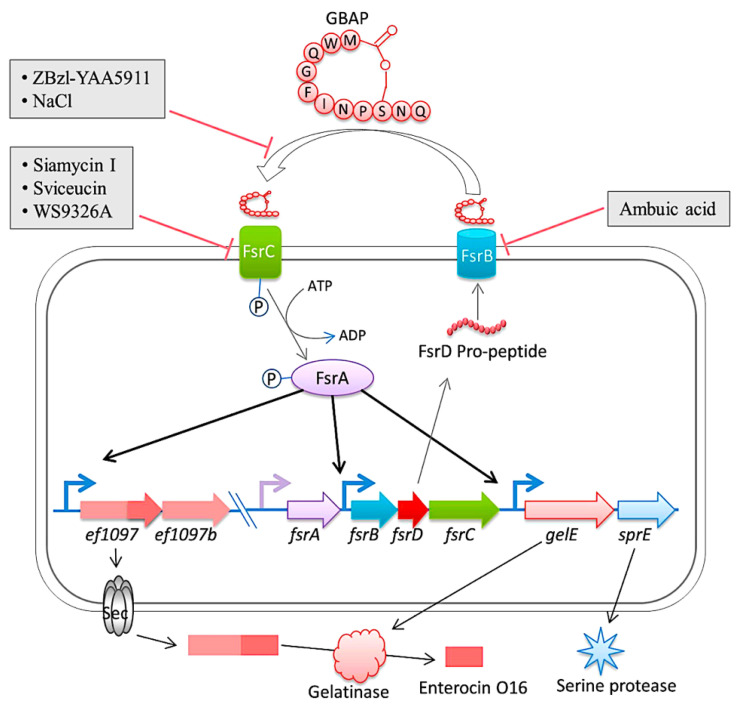
The diagram is a representation of the Fsr quorum sensing system and its regulation in *E. faecalis*. The system involves the export and processing of the FsrD propeptide (encoded by fsrD) to produce the small lactone gelatinase biosynthesis activating pheromone (GBAP) via FsrB. FsrC is part of a two-component regulatory system that reacts to extracellular GBAP by phosphorylating the intracellular response regulator FsrA, which in turn induces the expression of ef1097, ef1097b, the fsr locus, gelE (encoding a gelatinase) and sprE (encoding a serine protease). The pre-protein encoded by ef1097 is cleaved and transported via the Sec-dependent pathway and the precursor is further truncated by the gelatinase to form enterocin O16. The interaction of GBAP with FsrC is inhibited by ZBzl-YAA5911 in a competitive manner, whereas NaCl inhibits it in a concentration-dependent manner. The inhibition of FsrB activity is due to the presence of ambuic acid. Siamycin I, sviceucin, and WS9326A inhibited the phosphorylation of FsrC. From Ali et al. [151].

In a rabbit model of enterococcal endocarditis, the pathogenic capacity of vancomycin-resistant *E. faecalis* V583 and three isogenic protease mutants (ΔgelE, ΔsprE, and ΔgelE ΔsprE mutants) were compared [150]. Compared to V583 or the SprE(-) mutant, the bacterial load in the heart of the GelE(-) mutants (ΔgelE and ΔgelE ΔsprE mutants) was considerably reduced. A marked deposition of the fibrinous matrix layer and increased chemotaxis of inflammatory cells was also observed on aortic valves infected with GelE(-) mutants (ΔgelE and ΔgelE ΔsprE mutants). This suggests a role for proteolytic modulation of the immune response to *E. faecalis*. Furthermore, it was observed that GelE can degrade the anaphylatoxin complement C5a and that this proteolysis leads to reduced neutrophil recruitment in vitro, supporting a role for proteolytic modulation of the immune response to *E. faecalis*. In vivo, GelE-producing strains were observed to cause a decrease in heterophil migration at infected tissue sites, while SprE-producing strains did not show this effect. These results indicate that of the two enterococcal proteases, GelE is the most important in mediating the pathogenesis of endocarditis. Perez et al. published an important study in which the gene encoding gelatinase (gelE) was found to be under the control of the Fsr quorum sensing system, whose encoding genes (fsrA, fsrB, fsrC, and fsrD) are situated immediately upstream of gelE. Biofilm formation was prevented and gelatinase activity was suppressed in a derived mutant of *E. faecalis* V583 when a DNA fragment was integrated into the fsr locus. Sequence analysis revealed the presence of IS256 integrated into the fsrC gene at nucleotide position 321. It is worth noting that IS256 is also linked to biofilm formation in *Staphylococcus epidermidis* and *Staphylococcus aureus* [150] (Figure 5 [151]).

Enterococcal pathoadaptation to the endocardium is believed to be facilitated by the IS256 element, which causes gene inactivation and recombination. However, the regulation and activation mechanisms of IS256 remain poorly understood. To describe how chronic lytic phage infection leads to extensive amplification of IS256 in *E. faecalis* and how antibiotic exposure is associated with amplification of IS256 in *E. faecium* during clinical human infection, Kirsch et al. [152] recently applied an IS256-specific deep sequencing approach. Comparative genomics assessment revealed that IS256 is predominantly expressed in hospital-acquired enterococcal isolates. IS256 mobility in *E. faecalis* is transcriptionally regulated by multiple mechanisms, indicating tight control of IS256 activation in the absence of selective pressure. The results show that rapid genome-scale transposition in enterococci is driven by stressors such as phages and antibiotic load. IS256 diversification may thereby illustrate how evolutionary selection mediates enterococcal genome evolution, ultimately leading to the development of dominant nosocomial lineages threatening human health.

Brown et al. have recently reported in an experimental mouse model setting that peritoneal inoculation of *E. faecalis* can result in sub-endothelial microlesions in the heart [118,153]. The study also showed a strong immune response to the infection, indicating that different inoculation routes may result in varying outcomes for both the host and the bacteria. *E. faecalis* invades the vascular endothelium to enter myocardial tissue and induce cell death [118]. Notably, *E. faecalis* lacks homologs of pneumococcal surface adhesin CbpA, pneumolysin (ply), and pyruvate oxidase (spxB), suggesting the involvement of other factors. However, it can produce reactive oxygen species (ROS) [154]. ROS release by *E. faecalis* may therefore also be involved in cell death and microlesion development. One protein that has been found to affect *E. faecalis* cardiac microlesion formation is a disulphide bond-forming (Dsb) protein called DsbA. Thioredoxins, such as DsbA, play a crucial role in various bacterial fitness and pathogenicity factors, including biofilm formation, cell division, virulence, motility, cell wall synthesis, and growth. Proteins with a highly expressed CXXC active site motif interact with the free thiol groups of substrate cysteines, catalysing a disulphide linkage. Gram-positive bacteria have a lesser understanding of oxidative protein folding than gram-negative bacteria [155].

## 4. Point and Counterpoint

From a clinical point of view, the pathophysiology of IE is centred on the functional changes caused by bacterial damage to the cardiac valves. This process is generally believed to follow a foreseeable course: deployment of host factors at a site of endocardial surface injury or impairment, development of vegetations, valvular insufficiency, and decline in cardiac function. Staphylococci or streptococci are the most common causes of acute infective endocarditis in clinical practice, usually with a fast-moving, febrile course [12]. Chronic (subacute) IE, on the other hand, is more often related to a slowly developing, more insidious course with prodromal malaise and non-specific findings: oral streptococci and enterococci are the most likely pathogens in these instances [156]. For complex reasons previously discussed [60,157], although the incidence of bacterial endocarditis is generally steady or decreasing in modern health care systems, the proportion of cases due to enterococci has been on the rise [60,157].

From the 1970s onwards, a substantial proportion of both fundamental and clinical investigations in the endocarditis literature have suggested that physical injury to the vascular endothelium is a prerequisite for the active pathogenesis of IE. Most current frameworks assume an initial host immune reaction involving platelets, soluble components of the coagulation cascade, etc., with subsequent bacterial invasion of the emerging thrombus [158,159]. Upon close scrutiny of the historical references prior to 1975, however, IE has been described in a wide variety of animal experimental settings in the absence of such damage [160,161,162,163,164,165]. The researchers found that removing the endothelium prior to infection increased the rate of vegetation formation and reduced the number of animals required for the experiments. But this is simply an issue of convenience and efficiency, not biological need [1,166,167,168,169,170,171,172,173].

Therefore, while it is possible that pre-existing cardiac structural abnormalities or disorders of the cardiac endothelium in humans may increase the risk of bacterial colonisation and endocarditis, there is little evidence to suggest that overt endothelial surface disruption is necessary for bacterial colonisation, as previously reported [1,174]. However, even in previously published experimental studies in which pre-inoculation endothelial injury was not included, the process of bacterial invasion is still considered to rely on an existing host-derived thrombus as a precondition [156]. It is worth noting that certain pathogens can directly colonise the endothelial surface in certain circumstances [11]. In a recent study by Barnes et al. [13], it was reported that *E. faecalis* directly colonised the undamaged endothelial surface in a rabbit model system of endocarditis, without any obvious participation of host factors. Specifically, Barnes et al. discussed endothelial colonisation, which refers to the assembly of non-valvular microcolonies and biofilm formation as a bacterial mechanism for persistent infection, rather than classic frank valvular endocarditis. Further investigation of this aetiology is relevant, although there is no evidence to suggest that the attachment of enterococci to the valve surfaces is markedly distinct. Importantly, endothelial coverage and establishment of biofilm on valvular surfaces may be temporally distinct. This suggests that a suspected gastrointestinal source of enterococcal bacteraemia may progress through multiple steps before presenting with clinical signs of endocarditis [13,16].

The conventional endocarditis research and development studies show platelets and fibrin as the bare subendothelial components. The main question is how enterococci interact with the surface of normal cells. Jamet et al. [175] found that in the vasculature, enterococci may bind to circulating von Willebrand factor (vWF), similar to *Staphylococcus aureus* and *Streptococcus pneumoniae* [128,129,176,177]. Moreover, vWF is a crucial constituent of vertebrate haemostatic signalling pathways [178,179,180], and *E. faecalis* strain OG1RF contains virulence factors (ElrA) that seem to be involved in dealing with vWF domains [175]. This mechanism involves circulating von Willebrand factor (vWF) binding to free-floating bacteria. The bacteria then attach to surface-bound vWF on endothelial cells, which allows them to adhere to the cell surface. This process is believed to inhibit platelet recruitment and other responses of the host coagulation cascade by shrouding the bacteria in host vWF. Or, conversely, a previous paper report by Gaytán et al. [181] showed that a new adhesin that binds to sialic acid is crucial for infective endocarditis in several bacterial species. However, it is unclear how this relates to enterococcal endocarditis. Although host-factor interactions cannot be excluded in enterococcal IE, Barnes et al. [13,14,16] have shown that *E. faecalis* microcolonies form in a similar way in the vasculature and other non-circulatory disease settings, such as the murine gastrointestinal tract and in vitro polymer surfaces. This suggests the existence of another, perhaps more common, mechanism of adhesion. 

The potential for patients with enterococcal endocarditis to infect themselves through GI translocation would resolve several clinical problems in identifying the source of infection in many instances. Antibiotic and systemic stress can cause increased gut permeability to enterococci, which is a common occurrence in both outpatient and inpatient settings. Furthermore, in some endovascular infection models, there is no clear systemic, cell-mediated immune response observed, indicating that *E. faecalis* may evade the host immune system for extended periods. This complicates the establishment of definitive links between the onset of (potentially temporary) bacteraemia and endovascular colonisation. Further investigation is required to understand the potential and actual routes of patient self-infection in this area of research [182,183,184,185].

A multifaceted process is involved in the induction of enterococcal biofilm. It includes adherence to the surface, attachment, maturation of the microcolony, and the subsequent development of chronic disease. Despite extensive in vitro studies on the mechanisms of surface attachment, enterococcal virulence factors, plasmid exchange, and antibiotic resistance, their role in causing disease in vivo is still a matter of considerable debate. Furthermore, numerous laboratory-scale in vitro systems for studying biofilm formation have proven to be inconsistent with in vivo studies, indicating the need for further improvements. Additionally, the general mechanisms of biofilm formation in clinical disease states, including endocarditis, are understudied [182,183,184,185,186,187,188,189,190,191].

Over the past ten years, basic in vitro research has revealed that the genetic and physiological drivers of biofilm formation are likely to be highly variable between bacterial species: a universal biofilm inhibitor probably does not exist. Although some species may share similarities, it is also important to study the outliers, which include enterococci that have played a significant role for years. The genetic drivers involved in *E. faecalis* biofilm formation are shown in Table 2 [47,83,95,192,193,194,195,196,197,198,199,200].

In clinical settings, approximately half of enterococcal IE cases fail to identify a definitive source. This new framework suggests that prolonged persistence of enterococcal microcolonies on the cardiac endothelium may be consistent with a cloaked mechanism of enterococcal infection [183,201,202,203,204]. 

In vitro mechanistic studies provide evidence that platelets play a crucial role in the initial phase of infective endocarditis by constituting the first line of the immune response. This disease’s first phase is supported by the interaction of pathogens with platelets, making it a priority to counteract platelet antimicrobial activity. Experimental in vitro and animal models have suggested that aspirin can limit bacterial–platelet interactions, preventing vegetation development. These findings are promising. Clinical trial data on the outcome of patients with infective endocarditis treated with aspirin remain controversial. Contradictory findings cast a cloud of uncertainty over the benefit of antiplatelet agents in the prevention of infective endocarditis. In addition to aspirin, ticagrelor, an antagonist of the platelet receptor P2Y12, has been attributed with a therapeutic effect. This is due to its powerful antiplatelet activity and well-known antibacterial activity. In addition, a more recent study using a mouse model reported a significant capacity of ticagrelor to eradicate *Staphylococcus aureus* bacteraemia [205,206,207].

## Figures and Tables

**Table 1:** Pathogens in Allograft Infections in Non-Endocarditis and Comparison of Pathogens in Allograft Implants and Allograft Infections in Endocarditis.

Pathogen	Non-IE Allograft Infections		IE Pathogens at Allograft Implants		IE Pathogens at Allograft Infections	
	n * 22	No.%	n ^γ^ 46	No.%	n ^λ^ 42	No.%
*Staphylococcus aureus*	0 (0)		9 (20)		11 (26)	
*CoNS*	0 (0)		4 (8.7)		3 (7.1)	
Viridans group strep	10 (45)		5 (11)		7 (17)	
Enterococcus	0 (0)		7 (15)		3 (7.1)	
Others	3 (14)		5 (11)		5 (12)	
Pathogen not identified	3 (14)		9 (20)		5 (12)	
Other GPC	3 (14)		4 (8.7)		4 (9.5)	
Fungus	3 (14)		3 (6.5)		4 (9.5)	

Abbreviations: IE, Infective endocarditis; CoNS, coagulase-negative Staphylococci; GPC, Gram-positive cocci. * Data available for 22/30 non-IE patients with new allograft infection. ^γ^ Data available for 46 of 49 allograft recipients with IE at index procedure. ^λ^ Data available for 42 of 49 patients with IE with recurrent allograft infection.

**Table 2:** Genetic determinants that are involved in the formation of *E. faecalis* biofilm.

Gene/Locus	Protein/Function	Year of Publication
*srtC*	Sortase C/an enzyme that anchors surface proteins to the cell wall	2006 [47]
*atn*	Autolysin	2004 [83]
*salB*	Secretory antigen-like B/cell-shape determinant	2004 [83]
*bee*	Biofilm enhancer in Enterococcus/a putative cell wall-anchored protein	2006 [95]
*salA*	Secretory antigen-like A	2004 [83]
*bop*	Biofilm on plastic surface/a putative sugar-binding transcriptional regulator	2004 [96]
*gelE*	Secretory metalloprotease gelatinase E	2004 [8,83,97]
*dltA*	D-alanine lipoteichoic acid/D-alanine-D-alanyl carrier protein ligase	2006 [98]
*ebpA*, *ebpB*, *ebpC*	Endocarditis and biofilm-associated pili	2006 [47]
*ebpR*	Transcriptional regulator of ebpABC	2007 [99]
*epa* (*orfde4*)	Enterococcal polysaccharide antigen/a putative glycosyltransferase involved in polysaccharide synthesis	2004 [83]
*esp*	Enterococcal surface protein	2001, 2004 [94,100]
*etaR*	Enterococcal two-component system regulator	2004 [83]
*fsrA*, *fsrB*, *fsrC*	*E. faecalis* regulator/two-component quorum-sensing signal transduction system, regulates the expression of gelatinase and serine protease.	2004 [8,83,97]

## Data Availability

Not applicable.
